# Mechanoelectrical feedback in pulmonary vein arrhythmogenesis: Clinical challenges and therapeutic opportunities

**DOI:** 10.1002/joa3.12391

**Published:** 2020-06-29

**Authors:** Yen‐Yu Lu, Yao‐Chang Chen, Yung‐Kuo Lin, Shih‐Ann Chen, Yi‐Jen Chen

**Affiliations:** ^1^ Division of Cardiology Department of Internal Medicine Sijhih Cathay General Hospital New Taipei City Taiwan; ^2^ School of Medicine Fu‐Jen Catholic University New Taipei City Taiwan; ^3^ Department of Biomedical Engineering and Institute of Physiology National Defense Medical Center Taipei Taiwan; ^4^ Division of Cardiovascular Medicine Department of Internal Medicine Wan Fang Hospital Taipei Medical University Taipei Taiwan; ^5^ Cardiovacular Research Center Wan Fang Hospital Taipei Medical University Taipei Taiwan; ^6^ Heart Rhythm Center and Division of Cardiology Department of Medicine Taipei Veterans General Hospital Taipei Taiwan; ^7^ Graduate Institute of Clinical Medicine College of Medicine Taipei Medical University Taipei Taiwan

**Keywords:** atrial fibrillation, mechanoelectrical feedback, pulmonary vein

## Abstract

Mechanoelectrical feedback is an important factor in the pathophysiology of atrial fibrillation (AF). Ectopic electrical activity originating from pulmonary vein (PV) myocardial sleeves has been found to trigger and maintain paroxysmal AF. Dilated PVs by high stretching force may activate mechanoelectrical feedback, which induces calcium overload and produces afterdepolarization. These results, in turn, increase PV arrhythmogenesis and contribute to initiation of AF. Paracrine factors, effectors of the renin‐angiotensin system, membranous channels, or cytoskeleton of PV myocytes may modulate PV arrhythmogenesis directly through mechanoelectrical feedback or indirectly through endocardial/myocardial cross‐talk. The purpose of this review is to present laboratory and translational relevance of mechanoelectrical feedback in PV arrhythmogenesis. Targeting mechanoelectrical feedback in PV arrhythmogenesis may shed light on potential opportunities and clinical concerns of AF treatment.

## Graphical abstract

Mechanoelectrical feedback is an important factor in the pathophysiology of atrial fibrillation. Paracrine factors, effectors of the renin‐angiotensin system, membranous channels or cytoskeleton of pulmonary vein (PV) myocytes may modulate PV arrhythmogenesis directly through mechanoelectrical feedback or indirectly through endocardial/myocardial cross‐talk.

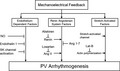

## 1 INTRODUCTION

Mechanoelectrical feedback refers to alterations in electrophysiological properties of cell or tissue as a result of changes in the loading conditions.^1^ Under pathological conditions or during mechanical perturbations, myocardial stretch results in increased vulnerability to arrhythmias by increasing volume, pressure, or direct distension of a muscle strip.^2^ Stretch‐activated mechanisms contribute to local or global changes in cardiac electrophysiology and intracellular calcium (Ca^2+^) handling.^3^, ^4^

Pulmonary veins (PVs) contain myocardial sleeves extending from the left atrium (LA),^5^ consisting a mixture of working cardiomyocytes and pacemaker cells.^6^ Ectopic electrical activities originating from PV myocardial sleeves have been found to both trigger and maintain paroxysmal atrial fibrillation (AF) in humans.^7^ Cellular mechanisms proposed for the generation of PV ectopy include increasing automaticity and triggered activity of PV cardiomyocytes.^8^, ^9^

Stretch‐induced mechanoelectrical feedback has been proven to regulate PV arrhythmogenesis.^10^ Stretch receptors have been found in the subendocardial tissues of LA and terminal portions of the PVs,^11^ Higher strain occurs in areas adjacent to PV trunks where atrial arrhythmias are most likely to occur.^12^ Patients with paroxysmal AF show dilatation of the orifices and proximal portions of their corresponding PVs.^13^ Dilated PVs have been reported to associate with high stretch level.^1^ PV dilatation provides not only structural alteration but also changes in electrical properties of the PVs,^14^ which may enhance PV arrhythmogenicity resulting in a higher incidence of AF development.^15^ Mechanoelectrical feedback in dilated PVs with a high stretch level may induce membrane depolarization and prolong action potential (AP) duration in isolated PV cardiomyocytes, which would alter refractoriness and dispersion and increase the vulnerability to AF.^2^, ^16^, ^17^

## 2 LABORATORY AND TRANSLATIONAL RELEVANCE OF MECHANOELECTRICAL FEEDBACK IN PV ARRHYTHMOGENESIS

Electrophysiological changes of mechanoelectrical feedback include (a) contour changes of cardiac AP, including shortening of AP duration, decrease in resting diastolic potential, and decrease maximum systolic AP amplitude; (b) development of early afterdepolarizations; and (c) ectopic beats originating from afterdepolarization in myocardium sustaining the greatest stretch.^18^

The spontaneous activity of PV cardiomyocytes is mainly regulated by Ca^2+^ homeostasis.^19^ The mechanoelectrical feedback increases Ca^2+^ transient to generate delayed and early afterdepolarization during shortening of AP duration.^10^, ^20^ Stretch‐dependent regulation of the Ca^2+^ system is operated via the mechanotransduction process. Stretch may induce Ca^2+^ overload and produce afterdepolarizations in cardiomyocytes.^21^ Its mechanisms involve many signaling cascades that target a diversity of intracellular Ca^2+^ sources. Stretch modifies cellular Ca^2+^ homeostasis by increasing the Ca^2+^ influx via stretch‐activated channels^22^ or the secondary effects of Na^+^ influx, followed by extrusion of Na^+^ accompanied by Ca^2+^ influx via sodium/calcium exchanger (NCX).^23^, ^24^ Moreover, some sarcolemmal voltage‐gated channels, such as L‐type Ca^2+^ current (*I*_Ca‐L_), have been reported to possess mechanosensitivity.^25^ Stretch appears to associate with changes to Ca^2+^ cycling with decreased *I*_Ca‐L_, which contributes to increased sarcoplasmic reticulum Ca^2+^ content despite unchanged or lower Ca^2+^ reuptake by sarcoplasmic reticulum Ca^2+^‐ATPase (SERCA) and increased diastolic Ca^2+^ leak.^26^

Acute stretch has been associated with conduction slowing and complex signal formation in the PV‐LA junction in human studies, and prolongation of refractory period in animal studies.^4^ The stretch increases PV firing rates and the incidence of spontaneous and triggered activities.^10^ Mechanical stretch through activation of stretch‐activated ion channel enhances PV spontaneous activity, contributing to phase 4 depolarization and leads to increase in firing rate.^27^ Activation of stretch‐activated channels can also affect both inward and outward ionic currents, resulting in shortening AP duration and increasing automaticity.^16^ Most voltage‐sensitive ion channels that give rise to the cardiac AP can be mechanically modulated. Furthermore, medications could affect the mechanosensitivity of ion channels.^28^

The effects of altered stretch on PV cardiomyocytes not only influence the internal machinery of cells, in part, via stretch‐activated channels,^29^ but also regulate actin filaments which modulate ion channel activity.^30^ Stretch‐induced myofilament Ca^2+^ release is also a strongly coupled form of mechanoelectrical feedback in cardiomyocytes.^31^ Release of Ca^2+^ from the myofilaments propagates through neighboring tissue by a combination of mechanical transduction, Ca^2+^ diffusion, and Ca^2+^‐induced Ca^2+^ release. This triggered detachment of Ca^2+^ from troponin C has been hypothesized leading toward extra‐systoles.^31^ In biopsies from patients with AF, re‐expression of α‐smooth muscle actin in endothelial cells surrounding the PVs contains stress fibers, which contributes to arrhythmogenicity.^32^ Cells lose their arrhythmic effects on cardiomyocytes after ablation of this cytoskeletal component.^33^ This suggests that stretch‐induced Ca^2+^ release by modulating actin filaments during structural remodeling may contribute to AF genesis.

## 3 TARGETING PV ARRHYTHMOGENESIS THROUGH STRETCH MODULATION

### 3.1 Endothelium‐dependent regulation

PVs contain endothelium and smooth muscle, which may produce nitric oxide (NO) through the enzyme eNOS or iNOS. Previous studies have shown that NO has important regulatory effects on the cardiovascular system.^34^ NO has been shown to regulate PV arrhythmogenesis through mechanoelectrical feedback,^35^ and reduce the triggered arrhythmias generated by Ca^2+^ overload.^36^ Mechanical stimulation of NO elevates the systolic Ca^2+^ transient and produces spontaneous Ca^2+^ sparks during diastole in myocytes contracting against a higher preload or afterload.^37^ Perioperative administration of nitroprusside (NO donor) during the rewarming period could prevent postoperative AF in patients undergoing myocardial revascularization, which suggests the anti‐AF effects of nitroprusside.^38^ Our previous study showed that nitroprusside could directly suppress spontaneous activity and inhibit delayed afterdepolarization with the decrease of transient inward currents in PV cardiomyocytes.^39^ These findings suggest that NO may play a role in PV arrhythmogenesis and become a specific target for pharmacological treatment of AF.

Endothelin appears to act as a mediator in the pathogenesis of hypertension and its complication resulting from mechanical loading. The major site of generation of endothelin‐1 is in endothelial cells.^40^ Endothelin‐1 is a potent vasoconstrictor that increases blood pressure and vascular tone. Endothelin‐1 modulates Ca^2+^ and potassium (K^+^) currents, and decreases PV spontaneous activity while increasing resting diastolic tension.^41^ Endothelin‐1 may have antiarrhythmic potential through its direct electrophysiological effects on PV cardiomyocytes, and is potentially a novel pharmacological agent against AF.

**FIGURE 1:**
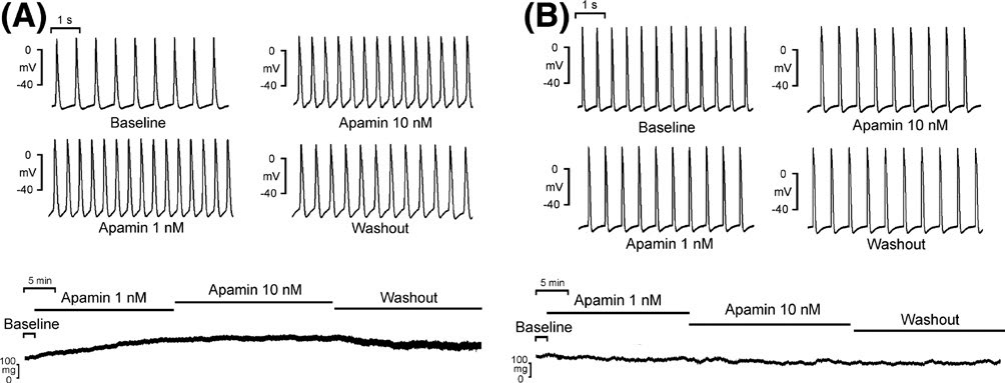
Effects of apamin on automaticity and vessel tone of pulmonary veins (PVs) with or without intact endothelium. A, With intact endothelium, apamin‐increased spontaneous activity and vessel tone in the PVs. B, Apamin decreased spontaneous activity, but did not change vessel tone in denudated PVs. Adopted from Ref. [44] with permission number of 4765180023726 and 4765180023726

Previous studies indicated small‐conductance Ca^2+^‐activated K^+^ (SK) channels plays an important role in maintaining vessel relaxation,^42^ while the endothelium is shown to release endothelium‐derived hyperpolarizing factors following activation of SK channels.^43^ Apamin (SK channel blocker) increased spontaneous activity and vessel tone in the PVs with a preserved endothelium, and reduced PV spontaneous activity without significantly affecting vessel tone when the endothelium was removed (Figure 1).^44^ These findings indicate that SK channel modulation may decrease vessel stretch and PV spontaneous activity.

### 3.2 The renin‐angiotensin system regulation

The renin‐angiotensin (Ang) system (RAS) plays a critical role in the pathophysiology of AF, which contributes to onset and maintenance of AF in paced animal models.^45^ The most active element of RAS is Ang II. The formation of Ang II involves two main steps: renin cleaves angiotensinogen to form Ang I, and angiotensin‐converting enzyme then cleaves Ang I to form Ang II. In addition, Ang I is hydrolyzed and converted by angiotensin‐converting enzyme into Ang (1‐7).^46^ Under pathophysiologic conditions, the local RAS may be more efficient in heart tissue than the systemic RAS. The local RAS may have a significant role in vasculature and cardiac myocytes.^47^

Mechanical stretch of cultured rat cardiac myocytes caused a rapid secretion of Ang II,^48^ and this autocrine production of Ang II may play a critical role in stretch‐induced arrhythmias. Ang II increases PV spontaneous activity and triggered activity as delayed afterdepolarizations, which may induce AF by increasing PV arrhythmogenic activity.^49^ Ang II receptor antagonists have been shown to prevent the occurrence of AF,^45^ and the mechanoelectrical feedback by decreasing stretch was proposed as one of the mechanisms.^50^ Losartan (Ang II receptor antagonist) could inhibit PV spontaneous activity. In addition, losartan could prevent or attenuate the proarrhythmic effects of Ang II in PV cardiomyocytes. These findings indicate that Ang II receptor antagonists have a direct antiarrhythmic effect through the reduction of the PV arrhythmogenic activity, and also work as an antagonist of Ang II to prevent AF.

Renin‐angiotensin system inhibition could be achieved by renin inhibitors, the first rate‐limiting step in RAS, which are thought to be more efficient than other inhibitors such as angiotensin‐converting enzyme or Ang II receptor antagonists. The renin inhibitor enalkiren has more effect on vasodilatation renal response compared to captopril.^51^ Aliskiren decreased *I*_Ca‐L_, but increased reverse‐mode NCX current, which reduces ${\text{Ca}}_{i}^{2+}$ transients, and SR Ca^2+^ content in PV cardiomyocytes. Aliskiren decreased PV diastolic tension, and reduced PV firing rate in a concentration‐dependent manner.^52^ These findings may reveal the antiarrhythmic potential of aliskiren.

Ang‐(1‐7) opposes the molecular and cellular effects of Ang II.^53^ Previous study has showed that Ang‐(1‐7) decreased *I*_Ca‐L_, late sodium current (*I*_Na‐Late_), and NCX current in PV cardiomyocytes.^54^ These might cause a reduction in ${\text{Ca}}_{i}^{2+}$ transients and SR Ca^2+^ content, and prevent ${\text{Ca}}_{i}^{2+}$ overload. The effects of Ang‐(1‐7) may reduce the PV spontaneous electric activity and triggered activity, which suggests the anti‐AF potential of Ang‐(1‐7).

### 3.3 Stretch‐activated channel regulation

Stretch‐activated ion channel blockers were shown to inhibit stretch‐induced changes in AP and afterdepolarizations,^55^ and decrease the vulnerability to AF.^56^ It was also reported that the stretch‐induced AF in perfused rabbit hearts was successfully inhibited by venom of the tarantula Grammostola spatulata which is a stretch‐activated ion channel blocker.^56^ The arrhythmogenic effects caused by high stretch levels in the PVs were reported to be attenuated by stretch‐activated ion channel blockers, gadolinium and streptomycin.^10^ The reversal of stretch‐induced shortening of AP duration by gadolinium also prevents the genesis of microreentrant circuits in the PVs. These findings indicate that stretch‐activated ion channel blockers could reduce AF genesis by suppressing the arrhythmogenic activity of the ectopic foci.

### 3.4 Stretch‐induced cytoskeleton modulation in PV arrhythmogenesis

**FIGURE 2:**
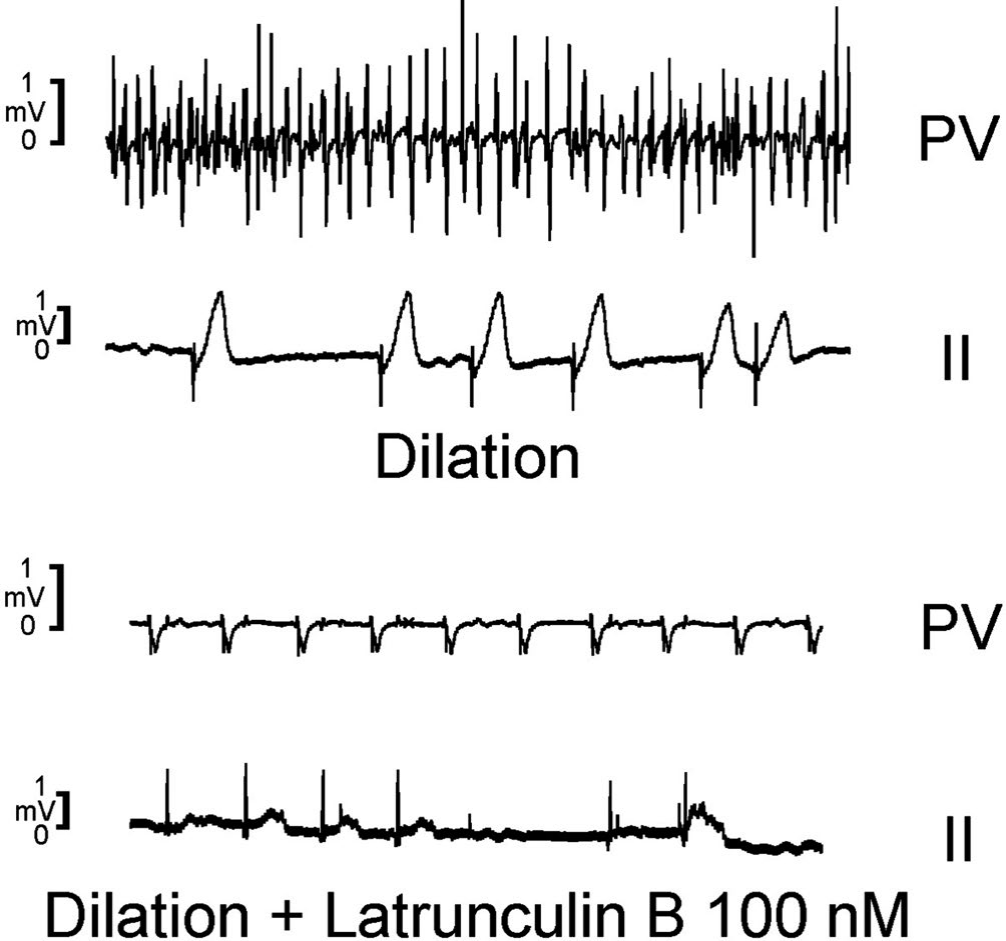
Effect of stretch and latrunculin‐B (Lat‐B) on pulmonary vein (PV) electrical activity in Langendorff‐perfused rabbit hearts. PV spontaneous electrical activity and bipolar atrial electrogram in atria under stretch with or without Lat‐B (100 nmol/L) treatment. Lat‐B (100 nmol/L) terminates PV burst firing in bipolar atrial electrogram. This figure was originally published in Lu YY, et al Clin Sci. 2016 with permission from the copyright holder^59^

Cells lose their arrhythmic effects on cardiomyocytes after pharmacological ablation of α‐smooth muscle actin‐containing stress fibers.^33^ This suggests that cytoskeletal rearrangement during structural remodeling may contribute to AF genesis. Mechanical stretch activates Ca^2+^ influx via stretch‐activated channels which are tightly regulated by the actin cytoskeleton.^57^ Actin polymerization could be inhibited by either sequestering the free actin monomer pool with drugs such as latrunculin‐B (Lat‐B).^58^ Lat‐B modulates stretch‐induced mechanoelectric feedback. Lat‐B decreases stretch‐induced large‐conductance Ca^2+^‐activated K^+^ channel current, as well as *I*_Ca‐L_, *I*_Na‐Late_, and NCX current, which may reduce the Ca^2+^_i_ transient amplitudes and the SR Ca^2+^ content. The decrease in *I*_Ca‐L_, *I*_Na‐Late_, and NCX current by Lat‐B may be related to the modulation of stretch‐induced changes, which may reduce PV arrhythmogenesis.^23^ Moreover, Lat‐B decreases PV spontaneous electrical activity (Figure 2).^59^ The antiarrhythmic effects of Lat‐B on the PVs indicated that actin polymerization may play a pivotal role in PV arrhythmogenesis during structural remodeling, and inhibition of actin polymerization could reduce AF inducibility and perpetuation. Therefore, the effects of Lat‐B on Ca^2+^ regulation may contribute to decreased PV spontaneous electrical activity and attenuated stretch‐induced PV arrhythmogenesis.

## 4 PROARRHYTHMIC EFFECTS OF INOTROPES VIA MECHANOELECTRICAL FEEDBACK

**FIGURE 3:**
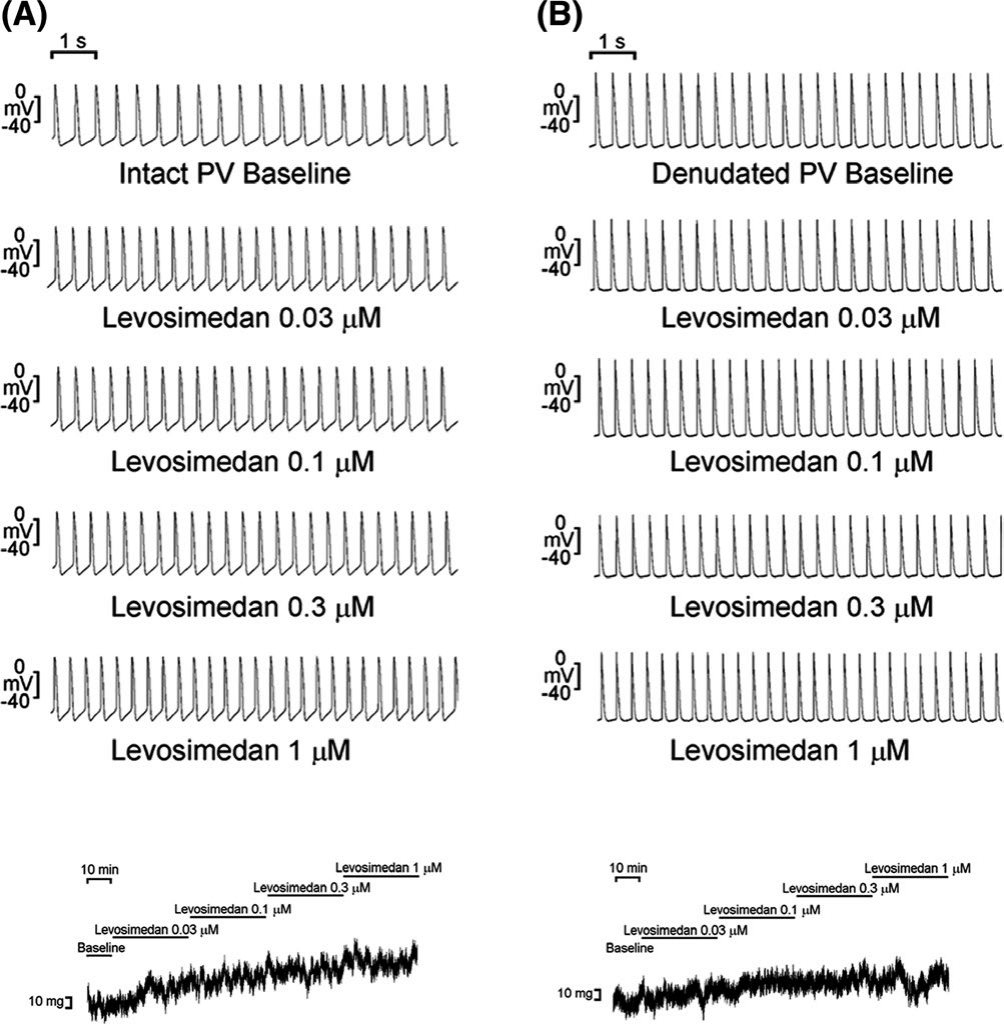
Effects of levosimendan on isolated pulmonary veins (PVs) with spontaneous activity and diastolic tension with or without intact endothelium. A, Levosimendan concentration dependently increases spontaneous activity and diastolic tension in the PVs with intact endothelium. B, Levosimendan did not change spontaneous activity and diastolic tension in denudated PVs. Adopted from Ref. [69] with permission number of 4765781439248

Conditions accompanied with mechanical stretch of the heart such as aging,^17^ high‐blood pressure, heart failure, and valvular dysfunction,^60^ may progress to overloaded myocardium and hence lead to decompensated myocardial function. These pathologic conditions are known to be risk factors for AF.^61^ Recently, higher NT‐proBNP (cardiac biomarker of myocardial stretch) was found to increase the risk of AF in people with chronic kidney disease.^62^ A variety of practical issues affect the development of new therapeutic approaches to AF. Previous studies have found that cardioactive agents such as isoproterenol or caffeine could increase PV arrhythmogenesis in chronic rapid pacing or advanced renal failure.^63^, ^64^ Agents such as levosimendan, milrinone, and digitalis have shown potential advantages in managing cardiac dysfunction.^65^ Digoxin was traditionally used in AF patients with heart failure because of its positive inotropic properties and a reduction in ventricular response. Digoxin increases automaticity and could produce virtually any type of cardiac dysrhythmia, including AF.^66^ Our previous study has found that digitalis may increase PV arrhythmogenesis through its effects on Ca^2+^ homeostasis and mechanoelectrical feedback because of increasing PV vascular tone through the enhanced contractility from its surrounding myocardium,^15^ which may contribute to AF occurrence. Similarly, milrinone has also been shown to increase PV arrhythmogenesis through mechanoelectrical feedback at least in part, potentially leading to the increased risk of AF.^67^ Levosimendan has vasodilatory properties; it facilitates the opening of adenosine triphosphate‐dependent K^+^ channels, or inhibits phosphodiesterase III.^68^ However, levosimendan increases PV tension through positive inotropic responses from the myocardial sleeve extending from the LA. The increasing PV diastolic tension by levosimendan may result in an acceleration of the spontaneous PV rate and burst firing through mechanoelectrical feedback (Figure 3).^69^ Positive inotropes administered to patients with heart failure may improve hemodynamics but are associated with an increased risk of AF.^70^, ^71^

## 5 IMPACT OF CATHETER ABLATION ON MECHANICAL FEEDBACK INDUCED PV ARRHYTHMOGENICITY

Catheter ablation has been established as a mainstay for the treatment of AF.^72^ Greater total PV volume and PV ostial area in paroxysmal AF patients are associated with AF recurrence after catheter ablation.^73^ The patients who underwent successful catheter ablation for AF showed an anatomical reverse remodeling with decrease in the ostial areas and the diameters of all four PVs measured 3 months after the procedure.^74^, ^75^ At 3 months follow‐up period, the mean ostial area was reduced by 14% in left superior PV, 19% in left inferior PV, 14% in right superior PV, and 16% in right inferior PV.^75^ Stress‐inducible microRNAs (miRs) plays a role in regulating myocardial remodeling. miR‐150 was downregulated in response to pressure overload and led to a reduction in cardiomyocyte cell size.^76^ As compared to participants without AF, patients with AF had lower plasma level of miR‐150, which was increased after catheter ablation.^77^ This finding suggests that catheter ablation for AF may reduce structural remodeling in AF patients via modulating transcriptomic profiles triggered by mechanical stretch. B‐type natriuretic peptide, a neurohormone secreted by cardiomyocytes in response to stretching, reduced to within normal range in the AF patients who had no AF recurrence after catheter ablation,^78^, ^79^ indicating that stretching force must be reduced after catheter ablation. Patients who kept sinus rhythm after ablation exhibited significantly higher levels of SERCA values compared to baseline, as well as compared to those who AF recurrence after ablation at 12‐month follow‐up.^79^ Accordingly, the stretch‐induced changes, such as Ca^2+^ handling, cytoskeletal modulation and RAS regulation, caused by PV dilatation are expected to be improved after successful ablation. However, plasma levels of renin and Ang II remain higher 6 months after catheter ablation.^80^ Therefore, successful ablation may not completely normalize stretch‐enhanced cytokines.

## 6 CONCLUSIONS

**FIGURE 4:**
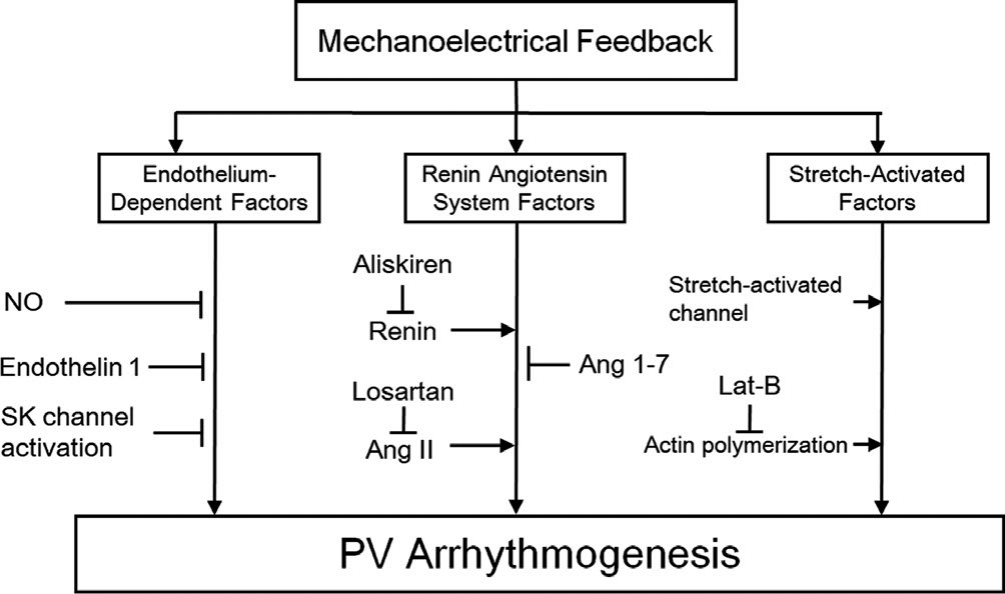
Targeting pulmonary vein (PV) arrhythmogenesis through modulating mechanoelectrical feedback. Ang, angiotensin; Lat‐B, latrunculin‐B; NO, nitric oxide; SK, small‐conductance calcium‐activated potassium

Pulmonary vein arrhythmogenesis is crucially dependent on the electrophysiological outcomes and Ca^2+^ handling characteristics of mechanoelectrical feedback. Multiple stretch‐activated mechanisms play important roles in PV mechanoelectrical feedback, which may contribute to the arrhythmogenic substrate in pathology. This should prompt the exploration of these mechanisms as potential novel therapeutic targets (Figure 4). Excessive mechanical stretch increases PV arrhythmogenesis because of mechanoelectrical feedback. A vicious circle ensues whereby one condition begets one another. Accordingly, targeting mechanoelectrical feedback in PV arrhythmogenesis may lead to potential opportunities for AF treatment.

## CONFLICT OF INTEREST

The authors declare no conflict of interest for this article.
